# Clustering cancer gene expression data by projective clustering ensemble

**DOI:** 10.1371/journal.pone.0171429

**Published:** 2017-02-24

**Authors:** Xianxue Yu, Guoxian Yu, Jun Wang

**Affiliations:** College of Computer and Information Science, Southwest University, Beibei, Chongqing, China; Ohio State University College of Medicine, UNITED STATES

## Abstract

Gene expression data analysis has paramount implications for gene treatments, cancer diagnosis and other domains. Clustering is an important and promising tool to analyze gene expression data. Gene expression data is often characterized by a large amount of genes but with limited samples, thus various projective clustering techniques and ensemble techniques have been suggested to combat with these challenges. However, it is rather challenging to synergy these two kinds of techniques together to avoid the curse of dimensionality problem and to boost the performance of gene expression data clustering. In this paper, we employ a projective clustering ensemble (PCE) to integrate the advantages of projective clustering and ensemble clustering, and to avoid the dilemma of combining multiple projective clusterings. Our experimental results on publicly available cancer gene expression data show PCE can improve the quality of clustering gene expression data by at least 4.5% (on average) than other related techniques, including dimensionality reduction based single clustering and ensemble approaches. The empirical study demonstrates that, to further boost the performance of clustering cancer gene expression data, it is necessary and promising to synergy projective clustering with ensemble clustering. PCE can serve as an effective alternative technique for clustering gene expression data.

## Introduction

With the rapid development of high-throughput biotechnologies, biologists can easily collect a large amount of gene expression data with low costs. Gene expression means that cells transfer the genetic information in deoxyribonucleic acid (DNA) into a protein molecule with biological activity through transcription and translation in life process [1]. Biologists measure expression levels under various specific experimental conditions to analyze gene functions, regulatory mechanisms and cancer subtypes [2, 3]. Given the wide applications of gene expression data in cancer diagnosis, gene treatments, prognosis and other domains [3–5], gene expression data analysis has been attracting increasing attention [1, 6].

Gene expression data can be presented as a matrix, with each row corresponding to a gene and each column representing a specified condition [7]. The specific conditions usually relate to environments, cancer types or subtypes and tissues. Each entry of the matrix corresponds to a numeric representation of the gene expression level under a given condition with respect to a particular gene. The first step of gene expression data analysis is to divide similar samples or genes into a group and dissimilar ones into different groups, which is recognized as gene expression data clustering. *k*-means was initially applied to group samples by assigning a sample to its nearest centroid, which is determined by the average of all samples in that group [8]. Eisen *et al*. [9] used average-link hierarchical clustering to cluster co-regulated genes of Yeast. Hierarchical clustering (HC) iteratively merges closest clusters by initializing each sample as a cluster or partitioning a huge cluster formed by all samples until a specified number of clusters is generated, and the distance between two clusters is defined as the average distance between samples of these two clusters. *k*-means and HC do not work well on high dimension gene expressional data, since the distance between samples becomes isometric when the gene dimensionality is very high [10].

With the development of modern molecular biological techniques (i.e., cDNA microarray, oligonucleotide microarray, gene sequencing), gene expression data is going to be with high dimensionality [11]. Gene expression data are usually characterized by thousands of genes but with very few samples. This characteristic often results in the curse of dimensionality problem [4] when grouping samples into different groups, and the distance between samples turns to be isometric [10]. Although these genes might be highly correlated, it is still rather difficult to determine the intrinsic dimensionality of these genes, so all genes are used for the clustering analysis. When clustering genes across samples, one may have clear knowledge of biological scenarios (i,e., a cell cycle), and thus we can control the construction of the sample space (i.e., taking time-course data over a cell cycle). On the other hand, when clustering samples (cancer patients), one has little knowledge about how to construct the gene space, since the relevant genes for a type of cancer are unclear [12]. For this reason, all the known genes are used for clustering, although it is widely recognized that only very few genes are relevant for a type of cancer. It is extremely challenging for unsupervised clustering to separate the samples, since many noisy (or irrelevant) genes will disturb the separation [13]. Particularly, traditional clusterings (*k*-means, HC) measure the similarity between samples by using all genes. Given that, these algorithms (i.e., *k*-means, HC) can not be effectively adopted to analyze high dimensional gene expression data.

In order to accurately group samples to their corresponding clusters, many clustering approaches have been proposed. For example, self-organizing feature map (SOM) [14], neural gas (NG) [15], PROCLUS [16], CLIQUE [17], local adaptive clustering (LAC) [18]. SOM [14] is a neural network model based on competitive learning, it uses neurons in the input layer to represent original data and a smaller number of neurons in the output layer (or competitive layer) to represent the compressed input data. Next, it employs neighborhood learning to adjust the weights between neurons in the input and output layers to approximate the underlying structure of input data. NG is similar to SOM, it utilizes a soft-max update rule to adjust the weights between neurons in the input and output layers. PROCLUS is a subspace-based clustering technique, it firstly uses a greedy algorithm to initialize centroids as apart as possible. Next, it searches an appropriate set of dimensions for each cluster to make the distance of a cluster to its centroid smaller than other set of dimensions. These found dimensions form the candidate subspace for the centroid and cluster. CLIQUE automatically searches subspaces with high density clusters. It partitions data space into cells, counts the number of points in each cell, and then takes the cell whose number of points greater than a predefined threshold as a dense unit. After that, it merges these dense units to form dense clusters. LAC optimizes the weight of each gene for each cluster and the weight reflects the relevance of the gene participating the cluster (or cancer subtype). However, these approaches depend on a single clustering algorithms and unstable, since they may suffer from noisy genes, improper setting of parameters and initial seeds.

Clustering ensemble, which fuses multiple clusterings into a consensus one, is shown to provide more stable clustering results and can avoid the risk of selecting a bad single clustering [19]. Multiple clusterings can be made by repeatedly running a single clustering algorithm with different initializations or input values of parameters [3, 16]. These base clusterings also can be derived from different clustering techniques [19, 20]. Therefore, various ensemble clustering techniques are also applied to analyze gene expression data [19, 21–24]. Genes are multi-functional and a gene can be relevant for more than one functional module (or cluster) [11, 25]. Given the nature of genes, researchers also use fuzzy clustering ensemble [26–28] to assign a gene (or sample) to several clusters.

It is recognized that only several features of high dimensional data contribute to a cluster or several clusters [18, 29]. Some projective clustering algorithms have been proposed to deal with high dimensional gene expression data [17, 18, 29]. However, it is difficult to integrate multiple projective clustering solutions, since most clustering ensemble techniques only address the multi-view nature of clustering and they do not tackle the high dimensional issue as well [30]. In other words, traditional clustering methods target at separately grouping genes or samples, and hence they only consider the relevance of a sample (or gene) belonging to a cluster. To bridge this gap, Gullo *et al*. [30] suggested a projective clustering ensemble (PCE) approach to take advantage of both projective clustering and ensemble clustering. PCE can not only take into account the relevance of a sample belonging to a cluster, but also the relevance of a gene contributing for the sample belonging to that cluster. These two relevances are called as *sample-to-cluster assignment* and *gene-to-cluster assignment*. Given the merits of PCE and characteristic of gene expression data, in this paper, we investigate the performance of PCE in clustering cancer gene expression data and quantitatively compare it with other related clustering algorithms [14, 18, 21, 31]. The experimental results show that PCE outperforms these comparing algorithms and PCE can serve as an effective technique for gene expression data analysis.

The rest of this paper is structured as follows. Section of related work briefly reviews the related clustering techniques for cancer gene expression data, followed with the basic principles of PCE. The cancer gene expression datasets and comparing methods are introduced in Section of experiment setup, followed with the Section of results and discussion.

## Related work

Single clustering algorithms were initially employed to cluster cancer gene expression data. Yeung *et al*. [32] proposed a model-based clustering method to cluster gene expression data. This method supposes that samples are generated by a finite mixture of underlying probability distributions, such as multivariate normal distributions, and then tries to divide samples into the best match distributions. Alizadeh *et al*. [33] applied hierarchical clustering to identify subtypes of diffuse large B-cell lymphoma. Although numerous single clustering algorithms have been widely applied in cancer gene expression data analysis, single clustering techniques often lack of accuracy, stability and robustness.

More recent techniques resort to ensemble clustering to group gene expression data and demonstrate stable and better performance than single clustering techniques. Ensemble clustering aggregates diverse clustering solutions from single clustering algorithm with different initializations, or from different clustering algorithms. Dudoit *et al*. [34] used Bagging [35] to generate diverse base clusterings, and then to aggregate these clusterings to assess the confidence of cluster assignments for individual samples. Smolkin *et al*. [36] used sub-sampling to generate multiple base clusterings and then fused these clusterings into a consensus one. Yu *et al*. [23] proposed a graph-based consensus clustering algorithm to estimate the underlying clusters of micro-array data. This algorithm obtains a set of base clustering solutions by repeatedly running subspace clustering or *k*-means, and results in multiple adjacent matrices between samples, each adjacent matrix corresponds to a clustering. Next, it constructs a graph by combining these adjacent matrices and uses normalized cut algorithm [37] to group samples. Domeniconi *et al*. [21] proposed a weighted similarity partitioning algorithm (WSPA) for clustering high dimensional gene expression data, WSPA takes LAC as the base clustering and to optimize the weights of genes for different clusters. After that, it adjusts the similarity between a sample and cluster centers based on the optimized weights of genes for ensemble clustering.

Fuzzy clustering techniques have also been applied to analyze cancer gene expression data [38]. Pedrycz *et al*. [28] proposed collaborative ensemble clustering based on fuzzy *c*-means [38]. Avogadri *et al*. [26] suggested a fuzzy ensemble clustering approach based on random projections of original high-dimensional gene expression data. Then, they applied fuzzy *k*-means algorithm on the projected data to generate multiple clusterings and combined these clusterings into a consensus one. Yu *et al*. [39] proposed a hybrid fuzzy ensemble clustering algorithm to cluster tumor bio-molecular data. Particularly, they employed affinity propagation clustering [40] to select representative genes and then applied multiple fuzzy clusterings on the samples with these selected genes for ensemble clustering. Yu *et al*. [31] suggested another adaptive fuzzy consensus clustering algorithm (RDCFCE) based on different clustering techniques. RDCFCE takes advantage of SOM [14] or NG [15] to project high dimensional genes into low grid dimension and takes these projected genes as representative genes, and then repeats multiple fuzzy clusterings on samples with respect to these representative genes for ensemble clustering. These ensemble clustering approaches improve the accuracy and robustness of single clustering algorithms on analyzing gene expression data, but they *only* take into account sample-to-cluster assignment and ignore the gene-to-cluster assignment.

More recently, co-clustering (or bi-clustering) [41–43] is also used to analyze gene expression data. Clustering only in the sample space may fail to discover the patterns that a set of samples exhibit similar gene expression behaviors only over a subset of genes. Co-clustering simultaneously performs clustering on both genes (or row) and samples (or column). One can obtain sets of genes that are co-regulated under a subset of samples via co-clustering algorithms. Liu *et al*. [44] proposed a network-assisted co-clustering to identify cancer subtypes. This method combines gene interaction network with gene expression profiles to simultaneously group genes and samples into biologically meaningful clusters. It can divide patients (samples) into different clinical subtypes and is robust to noise. Co-clustering ensemble is similar to clustering ensemble, it provides a framework to generate a more stable and robust consensus co-clustering by combining multiple base co-clusterings. Huang *et al*. [45] proposed a spectral co-clustering ensemble, which uses bipartite graph partition to leverage multiple base co-clusterings.

In this paper, we investigate the recently proposed PCE [30] and study its performance in clustering cancer gene expression data. Particularly, PCE can leverage the gene-to-cluster and sample-to-cluster assignments to disclose the underlying pattern of cancer gene expression data. In addition, PCE can integrate the advantages of ensemble clustering and projective clustering to mitigate the intrinsic issues (i.e., high dimensionality, few samples, many noisy genes) [46] of clustering gene expression data. Our experiments on various publicly available cancer gene expression data demonstrate that PCE can group samples more accurately than aforementioned related techniques (i.e., RDCFCE, WSPA).

## Projective clustering ensemble

**Fig 1:**
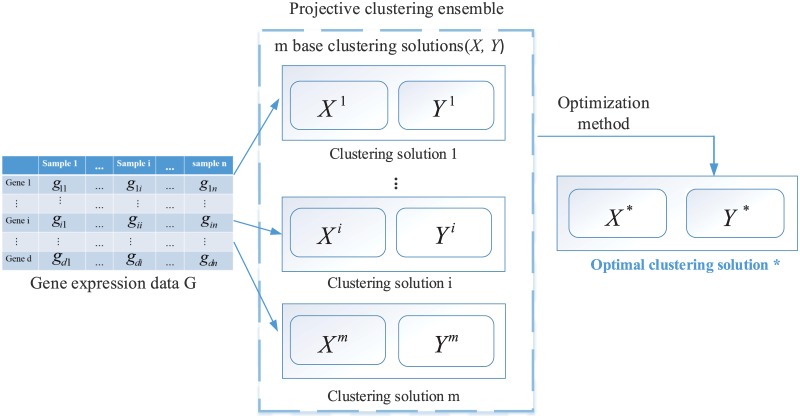
Framework of PCE. Base clustering solutions contain multiple sample-to-cluster assignments (*X*) and gene-to-cluster assignments (*Y*). *X* means the probability of samples belonging to clusters and *Y* means the relevance of genes to clusters. PCE aims to get the optimal X* and Y*.

Let matrix $G\in R^{d\times n}$ encode gene expression data for *d* genes with *n* samples, each row represents a gene, and each column represents a sample. Each entry of **G** corresponds to a numeric representation of the gene expression level under a given sample for a particular gene. PCE takes the information of gene-to-cluster assignment and sample-to-cluster assignment to formalize a final consensus clustering solution. If we separate samples into subtypes (or clusters), gene-to-cluster assignment means the probability that the gene is a relevant gene for a cluster, sample-to-cluster assignment means the probability of a sample belonging to that cluster. If we divide similar genes into a cluster, then gene-to-cluster assignment means the probability of a gene belonging to a particular cluster, sample-to-cluster assignment means the probability that the sample is a relevant sample for a cluster. In this paper, we aim to group similar samples into the same cluster and divide dissimilar ones into different clusters, based on expression profiles across *d* genes. Obviously, PCE is based on a set of diverse gene-to-cluster assignments and sample-to-cluster assignments. These assignments are generated by repeating projective clustering (i.e., LAC) *m* times with different initializations (or input values of parameters) to generate *m* clustering solutions, which serve as base clusterings for consensus clustering. Fig 1 illustrates the framework of PCE.

Suppose that *n* samples are divided into *k* clusters, different projective clustering solutions can have different values of *k*. $I_{l}=\left\{X^{l};Y^{l}\right\}$ is the *l*-th projective clustering solution, $X^{l}\in R^{k\times n}$ stores sample-to-cluster assignment and $Y^{l}\in R^{k\times d}$ encodes gene-to-cluster assignment. If the projective clustering is a hard clustering, then each entry of **X**^*l*^ is 1 or 0, otherwise each entry of **X**^*l*^ is between 0 and 1. PCE consists of many projective clustering solutions, $E=\{I_{1},I_{2},\ldots ,I_{m}\}$. We can write $X^{l}={\left[x_{1}^{l},\ldots ,x_{k}^{l}\right]}^{T}$ and each entry of $x_{k^{\prime }}^{l}\in R^{n}$ represents the probability of a sample belonging to the *k*′-th cluster, $\sum_{k^{\prime }=1}^{k}x_{k^{\prime }}^{l}=1$. Similarly, $Y^{l}={\left[y_{1}^{l},\ldots ,y_{k}^{l}\right]}^{T}$, each entry of $y_{k^{\prime }}^{l}\in R^{d}$ represents a gene’s relevance toward the *k*′-th cluster, $\sum_{d^{\prime }=1}^{d}y_{k^{\prime },d^{\prime }}^{l}=1$.

Given several clusterings from the same samples and a distance measure function, traditional ensemble clustering is to find a consensus clustering that minimizes the distance from all input clusterings [47]. For instance, given a ensemble E, consensus clustering is to optimize the following problem:

$$\begin{array}{c} I^{*}=\underset{I\subset E}{argmin}\;\psi \left(I,E\right) \end{array}$$ (1)

*ψ* is a distance function between clusterings. PCE is optimized from E with two requirements (sample-to-cluster assignment and gene-to-cluster assignment). PCE can be formulated as a two-objective optimization problem as follow:

$$\begin{array}{c} I^{*}=\underset{I}{argmin}\;\left\{\Psi_{s}\left(I,E\right),\Psi_{g}\left(I,E\right)\right\} \end{array}$$ (2)

Traditional ensemble clustering algorithms mainly focus on optimizing sample-to-cluster assignment ($\Psi_{s}\left(I,E\right)$). In contrast, PCE has to not only optimize sample-to-cluster assignment $\Psi_{s}\left(I,E\right)$, but also gene-to-cluster assignment $\Psi_{g}\left(I,E\right)$. To reach this target, Gullo *et al*. [30] adopted Pareto-based Multi-Objective Evolutionary Algorithms (MOEA) [48] to optimize Eq (2), and named MOEA based PCE as MOEA-PCE. However, since a large number of iterations is needed to get the final solution, MOEA-PCE is not so efficient that can not be applied to large scale datasets. To address this problem, Gullo *et al*. [30] employed an expectation maximization (EM) [49] style technique to alternatively optimize $\Psi_{s}\left(I,E\right)$ and $\Psi_{g}\left(I,E\right)$ in an iterative style, and they named EM based PCE as EM-PCE. Compared with MOEA-PCE, EM-PCE not only is more simple and efficient, but also has fewer input parameters. In this paper, we study EM-PCE for clustering cancer gene expression data.

Let $A^{l}\in R^{n\times d}$ store the probability of the intersection of events sample-to-cluster assignment (**X**^*l*^) and gene-to-cluster assignment (**Y**^*l*^) of the *l*-th projective clustering solution. This probability is equal to **X**^*l*^ joint with **Y**^*l*^ under the assumption of independence between two events. $A_{n^{\prime },d^{\prime }}^{l}=\sum_{k^{\prime }=1}^{k}X_{k^{\prime },n^{\prime }}^{l}Y_{k^{\prime },d^{\prime }}^{l}$ measures the relevance of the *d*′-th gene to the *n*′-th sample in the *l*-th clustering. We define $\Lambda \in R^{n\times d}$, whose entry $\Lambda_{n^{\prime },d^{\prime }}=\frac{1}{m}\sum_{l=1}^{m}\sum_{k^{\prime }=1}^{k}(X_{k^{\prime },n^{\prime }}^{l}Y_{k^{\prime },d^{\prime }}^{l}$) corresponds to the probability $Pr(A_{n^{\prime },d^{\prime }}|E)$ of the relevance **A**_*n*′, *d*′_, given the information available from projective ensemble E. The objective function of EM-PCE is defined as an error minimization criterion that takes into account both sample-to-cluster assignment and gene-to-cluster assignment. For any candidate consensus solution $I^{*}\in E$, the error is defined as $R_{k^{\prime },n^{\prime }}=\sum_{d^{\prime }=1}^{d}{\left(Y_{k^{\prime },d^{\prime }}^{*}-\Lambda_{n^{\prime },d^{\prime }}\right)}^{2}$, **R**_*k*′, *n*′_ reflects how well $Y_{k^{\prime }}^{*}$ in the candidate $I^{*}$ complies with Λ_*n*′_ of sample *n*′ within cluster *k*′ based on the information from E. Taking into account the error of all samples within clusters of the candidate $I^{*}$, EM-PCE can be reformulated as follows:

$$\begin{array}{c} I^{*}=argmin\;\Theta \left(X^{*},Y^{*},E\right) \end{array}$$ (3)

$$\begin{array}{c} \begin{array}{cc} s.t & \sum_{k^{\prime }=1}^{k}X_{k^{\prime },n^{\prime }}^{*}=1,\forall n^{\prime }\in \left\{1,\cdots ,n\right\},\\ & 0⩽X_{k^{\prime },n^{\prime }}^{*}⩽1,\forall n^{\prime }\in \left\{1,\cdots ,n\right\},\;\forall k^{\prime }\in \left\{1,\cdots ,k\right\}\\ & \sum_{d^{\prime }=1}^{d}Y_{k^{\prime },d^{\prime }}^{*}=1,\forall k^{\prime }\in \left\{1,\cdots ,k\right\},\\ & 0⩽Y_{k^{\prime },d^{\prime }}^{*}⩽1,\forall d^{\prime }\in \left\{1,\cdots ,d\right\},\;\forall k^{\prime }\in \left\{1,\cdots ,k\right\} \end{array} \end{array}$$ (4)

$$\begin{array}{c} \Theta \left(X^{*},Y^{*},E\right)=\sum_{k^{\prime }=1}^{k}\sum_{n^{\prime }=1}^{n}{\left(X_{k^{\prime },n^{\prime }}^{*}\right)}^{\alpha }\sum_{d^{\prime }=1}^{d}{\left(Y_{k^{\prime },d^{\prime }}^{*}-\Lambda_{n^{\prime },d^{\prime }}\right)}^{2} \end{array}$$ (5)

*α* > 1 is an integer that ensures X* ∈ [0, 1] instead of {0, 1}. Eqs (3–5) can be solved by the conventional Lagrange multipliers method, considering the relaxed problem obtained by temporarily dropping the inequality constraints ($X_{k^{\prime },n^{\prime }}^{*}\geq 0$ and $Y_{k^{\prime },d^{\prime }}^{*}\geq 0$) in Eq (4). Eq (3) can be relaxed and solved as follow:

$$\begin{array}{c} \Theta_{\lambda }\left(X^{*},Y^{*},E\right)=\Theta \left(X^{*},Y^{*},E\right)+\sum_{n^{\prime }=1}^{n}\lambda_{n^{\prime }}^{\prime }\left(\sum_{k^{\prime }=1}^{k}X_{k^{\prime },n^{\prime }}^{*}-1\right)+\sum_{k^{\prime }=1}^{k}\lambda_{k^{\prime }}^{\prime \prime }\left(\sum_{d^{\prime }=1}^{d}Y_{k^{\prime },d^{\prime }}^{*}-1\right) \end{array}$$ (6)

To optimize X*, we assume Y* as a constant, and compute the optimal X* as follow:

$$\begin{array}{c} \frac{\partial \Theta_{\lambda }}{\partial X_{k^{\prime },n^{\prime }}^{*}}=\alpha {\left(X_{k^{\prime },n^{\prime }}^{*}\right)}^{\alpha -1}\sum_{d^{\prime }=1}^{d}{\left(Y_{k^{\prime },d^{\prime }}^{*}-\Lambda_{n^{\prime },d^{\prime }}\right)}^{2}+\lambda_{n^{\prime }}^{\prime }=0 \end{array}$$ (7)

$$\begin{array}{c} \frac{\partial \Theta_{\lambda }}{\partial \lambda_{n^{\prime }}^{\prime }}=\sum_{k^{\prime }=1}^{k}X_{k^{\prime },n^{\prime }}^{*}-1=0 \end{array}$$ (8)

Combining Eqs (7) and (8), we can get the optimal **X**_*k*′, *n*′_:

$$\begin{array}{c} X_{k^{\prime },n^{\prime }}^{*}={\left[\sum_{k^{\prime \prime }=1}^{k}{\left(\frac{R_{k^{\prime },n^{\prime }}}{R_{k^{\prime \prime },n^{\prime }}}\right)}^{\frac{1}{\alpha -1}}\right]}^{-1} \end{array}$$ (9)

Similarly, we can fix X* and optimize Y*. The optimal Y* is computed as:

$$\begin{array}{c} \frac{\partial \Theta_{\lambda }}{\partial Y_{k^{\prime },d^{\prime }}^{*}}=2\sum_{n^{\prime }=1}^{n}{\left(X_{k^{\prime },n^{\prime }}^{*}\right)}^{\alpha }\left(Y_{k^{\prime },d^{\prime }}^{*}-\Lambda_{n^{\prime },d^{\prime }}\right)+\lambda_{k^{\prime }}^{\prime \prime }=0 \end{array}$$ (10)

$$\begin{array}{c} \frac{\partial \Theta_{\lambda }}{\partial \lambda_{k^{\prime }}^{\prime \prime }}=\sum_{d^{\prime }=1}^{d}Y_{k^{\prime },d^{\prime }}^{*}-1=0 \end{array}$$ (11)

Combining the Eqs (10) and (11), we can get the optimal **Y**_*k*′, *n*′_ as:

$$\begin{array}{c} Y_{k^{\prime },d^{\prime }}^{*}=\frac{\sum_{n^{\prime }=1}^{n}{\left(X_{k^{\prime },n^{\prime }}^{*}\right)}^{\alpha }\Lambda_{n^{\prime },d^{\prime }}}{\sum_{n^{\prime }=1}^{n}{\left(X_{k^{\prime },n^{\prime }}^{*}\right)}^{\alpha }} \end{array}$$ (12)

EM-PCE iteratively optimizes X* with Y* fixed and then optimizes Y* with X* fixed until convergence. In this way, we can get the final clustering solution of EM-PCE.

## Experiment setup

### Comparing methods and cancer gene expression datasets

To comparatively investigate the performance of EM-PCE on clustering cancer gene expression data, we take RDCFCE [31], WSPA [21], LAC [18], SOM [14], hierarchical clustering (HC) [8], *k*-means [9] as comparing methods. HC and *k*-means are two widely used traditional clustering methods. SOM and LAC are single clustering algorithms and their effectiveness is validated on clustering high dimensional data. RDCFCE is a fuzzy ensemble clustering approach. RDCFCE uses SOM [14] to project high dimensional genes into low grid dimension and takes the projected genes as representative genes. After that, it generates base clustering solutions (sample-to-cluster assignment) by repeating fuzzy *k*-means on samples with respect to these representative genes. WSPA is a weighted ensemble clustering algorithm, it employs LAC [18] with different input values of parameters to generate multiple base clusterings, but it only considers the sample-to-cluster assignments. EM-PCE also uses LAC to produce multiple base clusterings, it takes into account both the sample-to-cluster assignments and gene-to-cluster assignments.

We perform experiments on eight publicly available cancer gene expression datasets. Table 1 provides the brief description of these datasets. Breast includes four subtypes of tumors: 13 estrogen receptor (ER) + lymph node (LN) + tumors samples, 12 ER—LN + tumors samples, 12 ER + LN- tumors samples, and 12 ER—LN—tumors samples. DLBCLA includes three subtypes of diffuse large B cell lymphoma: ‘oxidative phosphorylation’ (49 samples), ‘B-cell response’ (50 samples), and ‘host response’ (42 samples). Leukemia includes six prognostic important leukemia subtypes: T-lineage acute lymphoblastic leukemia (ALL) (43 samples), E2A-PBX1 (E2A) (27 samples), BCR-ABL (BCR) (15 samples), TEL-AML1 (TEL) (79 samples), MLL rearrangements (20 samples) and ‘hyperdiploid>50’ chromosomes (Hyperdiploid) (64 samples). NovartisBPLC is composed of four distinct cancer types: breast (26 samples), prostate (26 samples), lung (28 samples), and colon (23 samples). Pomeroy2002v2 consists of four cancer types and one normal tissue: medulloblastomas (10 samples), malignant gliomas (10 samples), atypical teratoid/rhabdoid tumours (10 samples), primitive neuroectodermal tumours (8 samples), and normal tissues (4 samples). Ramaswamy2001 contains 190 samples, which are categorized into fourteen tumors subtypes: breast adenocarcinoma (11 samples), prostate adenocarcinoma (10 samples), lung adenocarcinoma (11 samples), colorectal adenocarcinoma (11 samples), lymphoma (22 samples), melanoma (10 samples), bladder transitional cell carcinoma (11 samples), uterine adenocarcinoma (10 samples), leukemia (30 samples), renal cell carcinoma (11 samples), pancreatic adenocarcinoma (11 samples), ovarian adenocarcinoma (11 samples), pleural mesothelioma (11 samples), central nervous system (20 samples). Risinger-2003 contains four subtypes: serous papillary (13 samples), clear cell (3 samples), endometrioid cancers (19 samples), and age-matched normal endometria (7 samples). Su2001 includes ten distinct types of carcinomas: prostate (26 samples), bladder/ureter (8 samples), breast (26 samples), colorectum (23 samples), gastroesophagus (12 samples), kidney (11 samples), liver (7 samples), ovary (27 samples), pancreas (6 samples), and lung (28 samples). From Table 1, we can easily observe that the number of involved samples is much smaller than the number of genes. These datasets cover different types (or subtypes) of cancers, and they can be collected from the reference alongside the dataset in Table 1. The ground-truth subtypes of these cancer gene expression datasets are known. In this way, we can compare the clustering results made by these comparing methods with the known ground-truths.

**Table 1:** Eight cancer gene expression datasets.

Dataset	Source	#Subtypes(*k*)	#Samples(*n*)	#Genes(*d*)
Breast	[50]	4	49	1213
DLBCLA	[50]	3	141	661
Leukemia	[22]	6	248	985
NovartisBPLC	[22]	4	103	1000
Pomeroy2002v2	[51]	5	42	1379
Ramaswamy2001	[51]	14	190	1363
Risinger2003	[51]	4	42	1771
Su2001	[51]	10	174	1571

#Subtypes is the number of cancer subtypes (or clusterings), #Sample is the number of samples, and #Genes is the number of genes.

### Evaluation metrics

Various evaluation metrics can be used to evaluate the quality of clustering. In this paper, we adopt three widely used external metrics: Rand index (RI) [52], Adjusted Rand index (ARI) [53] and Normalized Mutual Information (NMI) [54]. Suppose the ground truth clusters of *n* samples in $G\in R^{d\times n}$ are $C=\{c_{1},\ldots ,c_{k}\}$, clusters produced by a clustering method are $C^{\prime }=\left\{c_{1}^{\prime },\ldots ,c_{k^{\prime }}^{\prime }\right\}$. In this study, we take subtypes of a cancer or different cancer types as the ground-truth clusters. Since the ground-truth clusters are known, we can use external evaluation metrics (RI, ARI, NMI) to measure the difference between the clustering results and the ground-truths, and thus to quantitatively compare the performance of these methods.

Let *μ*_1_ represent the number of pairs of samples that are both in the same cluster of C and also both in the same group of $C^{\prime }$, *μ*_2_ represent the number of pairs of samples that are in the same cluster of C but in different groups of $C^{\prime }$, *μ*_3_ represent the number of pairs of samples that are in the different clusters of C but in the same group of $C^{\prime }$, *μ*_4_ represent the number of pairs of samples that are in different clusters of C and in different groups of $C^{\prime }$. RI measures the percentage of correct partitions, a lager RI value indicates a more satisfactory clustering solution. RI is defined as follow:

$$\begin{array}{c} RI=\frac{\mu_{1}+\mu_{4}}{\mu_{1}+\mu_{2}+\mu_{3}+\mu_{4}} \end{array}$$ (13)

ARI is an enhanced metric of RI. Suppose *n* is the total number of samples, *n*_*i*_ is the number of samples in the cluster *c*_*i*_, *n*_*j*_ is the number of samples in the cluster $c_{j}^{\prime }$, *n*_*ij*_ is the number of samples which belongs to cluster *c*_*i*_ and cluster $c_{j}^{\prime }$. ARI is defined as:

ARI=∑i=1k∑j=1k′nij2-q312(q1+q2)-q3q1=∑i=1kni2,q2=∑j=1k′nj2,q3=2q1q2m(m-1) (14)

NMI is defined as follows:

$$\begin{array}{c} NMI\left(C,C^{\prime }\right)=\frac{2*I(C,C^{\prime })}{H\left(C\right)+H\left(C^{\prime }\right)} \end{array}$$ (15)

where $I(C,C^{\prime })$ is the mutual information between C and $C^{\prime }$, and H(C) is the entropy of C. $I(C,C^{\prime })$ and H(C) are defined as follow:

$$\begin{array}{c} I\left(C,C^{\prime }\right)=\sum_{i=1}^{k}\sum_{j=1}^{k^{\prime }}p\left(c_{i},c_{j}^{\prime }\right)log_{2}\left(\frac{p(c_{i},c_{j}^{\prime })}{p\left(c_{i}\right)p\left(c_{j}^{\prime }\right)}\right) \end{array}$$ (16)

$$\begin{array}{c} H\left(C\right)=-\sum_{i=1}^{k}p\left(c_{i}\right)log_{2}p\left(c_{i}\right) \end{array}$$ (17)

where $p(c_{i},c_{j}^{\prime })$ is the joint probability distribution of *c*_*i*_ and $c_{j}^{\prime }$. If cluster *c*_*i*_ contain *n*_*i*_ samples, then *p*(*c*_*i*_) = *n*_*i*_/*n*. $I(C,C^{\prime })$ measures the statistical information shared by two clusterings. NMI is always between 0 and 1. If NMI = 1, the predicted solution is the same as the ground truth solution, and a larger NMI indicates better clustering solution.

## Result and discussion

### Result on clustering synthetic datasets

**Fig 2:**
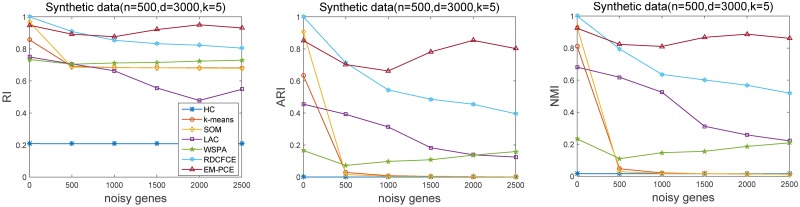
Accuracy(RI, ARI, NMI) on synthetic data. Noisy genes is the number of noisy genes in the synthetic data. RI, ARI and NMI reflect the performance of seven comparing methods under different numbers of randomly injected noisy genes. EM-PCE generally has higher accuracy than other methods on RI, ARI and NMI.

To better explain the curse of dimensionality and evaluate the effectiveness of these comparing methods, we firstly test these methods on synthetic gene expression datasets. The synthetic datasets are generated from normal distribution according to the mean and variance estimated from the gene expression profiles of T-lineage acute lymphoblastic leukemia (ALL), E2A-PBX1, BCR-ABL, TEL-AML1 and MLL rearrangements subtypes in the Leukemia cancer dataset. Particularly, these five clusters are generated by normal distribution N(1.3851 0.2337), N(1.2287 0.1630), N(1.3252 0.2806), N(1.2649 0.2225) and N(1.2016.5 0.2856) with 3000 genes (or features), and each cluster has only 100 samples. To make the synthetic datasets more realistic, we randomly injected noisy genes, each of which is a random numeric value between the minimum and maximum expression levels of the expression data. The number of noisy genes is set to 0, 500, …, 2500. This simulation process is also used in [22, 44]. In this way, six synthetic datasets are generated with different number of randomly injected noisy genes. We apply these clustering methods on these synthetic datasets. For each synthetic dataset, we perform ten independent runs and report the average and variance values of RI, ARI and NMI. In the experiments, the parameters of EM-PCE are *m* (the number of projective clustering solutions) and *α* (controlling the softness of sample-to-cluster assignment). *m* and *α* are are set as 100 and 2, respectively. EM-PCE generates base clustering solutions by repeatedly running LAC with 1/*h* = 1, …, *m*. In LAC, parameter *h* controls how much the distribution of weight deviating from the uniform distribution, we set *h* = 2 as suggested in [18]. The number of base clustering solutions in RDCFCE and WSPA is fixed as 100, too. Fig 2 gives the results of comparing methods on the synthetic datasets under evaluation metrics RI, ARI and NMI.

From this figure, we can observe that HC cannot correctly group samples into respective clusters, even though no noisy genes are injected at the beginning. That is because HC is very sensitive to redundant and noisy features and HC uses all the genes to measure the similarity between samples. This fact shows HC is not suitable for high-dimensional data clustering. When 500 or more noisy genes are injected, the accuracy of *k*-means and SOM decrease sharply. *k*-means randomly selects initial cluster centroids, because of noisy genes, a sample is not assigned to its ground truth nearest centorid. SOM maps the high gene dimension to low grid dimension, but it can not distinguish noisy genes. So its accuracy also downgrades. The accuracy of LAC decreases relatively smaller than HC, *k*-means and SOM. That is because LAC assigns genes with weights to indicate their importance and reduces the interference of noisy genes. Relevant genes are assigned with large weights and irrelevant ones (or noisy genes) are assigned with small (or zero) weights. These synthetic datasets have a large amount of genes, but a few of them are relevant for identifying the subtypes of samples. Since LAC is a single clustering solution, it is not robust to noisy genes. These observations indicate the necessity of ensemble clustering.

WSPA and DRCFCE are ensemble clustering methods, they are more robust to noisy genes than single clustering methods (*k*-means, HC, SOM). But WSPA and DRCFCE take information of many sample-to-cluster assignments to obtain the final clustering, they can not separate the samples well when a large amount of noisy genes are injected. When no noisy genes are injected, all the genes are relevant, EM-PCE does not show advantage than RDCFCE. The performance of EM-PCE is stable when the noisy genes are injected, but the performance of DRCFCE continuously decreases when more noisy genes are injected. The possible reason is that RDCFCE maps the gene-dimension to a low grid dimension by SOM, but SOM cannot distinguish noisy genes. In the real gene expression data, the relevant genes are usually very few. So EM-PCE is a more effective clustering method than RDCFCE and WSPA.

Compared with WSPA and DRCFCE, EM-PCE has higher accuracy when noisy genes are injected and is more robust to noisy genes. EM-PCE takes information from both gene-dimension and sample-dimension of many projective clustering solutions, and tries to find the optimal sample-to-cluster assignment and gene-to-cluster assignment. EM-PCE successfully groups samples under different numbers of noisy genes, and the grouped samples belonging to the same cluster have the similar gene expression profiles over a subset of genes, instead of all the genes. These investigations on synthetic datasets indicate that EM-PCE is a competitive clustering method for gene expression data analysis.

### Result on clustering real cancer gene expression data

We compare the performance of EM-PCE with *k*-means, HC, SOM, LAC, RDCFCE and WSPA on different cancer gene expression datasets. For each dataset and each comparing algorithm, we perform ten independent runs and report the average and variance values of RI, ARI and NMI. The average and variance reflect the accuracy and stability of an algorithm, respectively. For EM-PCE, we set *m* = 100 and *α* = 2. For LAC, the parameter *h* controls how much the distribution of weight deviating from the uniform distribution, as suggested by Domeniconi *et al*. [18], we set *h* = 2. The number of base clustering solutions in RDCFCE and WSPA is set as 100.

Tables 2 (RI), 3 (ARI) and 4 (NMI) are the results of these comparing approaches on eight gene expression datasets. In the table, the data in **boldface** is the statistical significantly best (or comparable best) results, and the significance is assessed by pairwise *t*-test at 95% level. We also use Wilcoxon’s signed-rank test [55, 56] (at 95% level) to compare the performance of these comparing methods across all the datasets, the *p*-value are all smaller than 0.004, except that for WSPA is 0.052. From Table 2, we can see that EM-PCE achieves better performance than other approaches on six out of eight datasets, which are Breast, DLBCLA, Leukemia, NovartisBPLC, Ramaswamy2001 and Su2001. Table 3 shows that EM-PCE outperforms other approaches on five out of eight datasets, which are Breast, DLBCLA, Leukemia, NovartisBPLC and Su2001. Table 4 shows that EM-PCE outperforms other approaches on three out of eight datasets, which are Breast, DLBCLA, Leukemia. These experimental results demonstrate that EM-PCE is an effective clustering technique for cancer gene expression data.

**Table 2:** RI (average and standard deviation) of HC, *k*-means, SOM, LAC, WSPA, RDCFCE and EM-PCE on eight gene expression datasets.

Dataset	HC	*k*-means	SOM	LAC	WSPA	RDCFCE	EM-PCE
Breast	0.3605(0)	0.6707(0.0055)	0.7046(0.0002)	0.6578(0.0016)	0.6895(0.0002)	0.6678(0.0002)	**0.7656(0.0007)**
DLBCLA	0.3424(0)	0.6098(0.0001)	0.6474(0.0001)	0.8898(0.0058)	0.8298(0.0003)	0.7086(0.0000)	**0.9528(0.0002)**
Leukemia	0.2408(0)	0.9346(0.0003)	0.8993(0.0019)	0.9370(0.0009)	0.8735(0.0003)	0.8476(0.0000)	**0.9777(0.0000)**
NovartisBPLC	0.6244(0)	0.8055(0.0078)	0.8604(0.0001)	0.9255(0.0046)	0.9587(0.0001)	**0.9802(0.0000)**	**0.9802(0.0000)**
Pomeroy2002v2	0.4425(0)	0.8262(0.0002)	0.8466(0.0005)	0.7168(0.0168)	**0.8990(0.0001)**	0.8188(0.0000)	0.8247(0.0000)
Ramaswamy2001	0.1887(0)	0.7090(0.0015)	0.8434(0.0001)	0.7558(0.0032)	0.9019(0.0000)	0.8318(0.0007)	**0.9124(0.0003)**
Risinger2003	0.3612(0)	0.5949(0.0079)	0.6906(0.0001)	0.6159(0.0037)	0.6871(0.0001)	**0.7556(0.0000)**	0.7153(0.0009)
Su2001	0.2378(0)	0.7464(0.0024)	0.7737(0.0003)	0.8032(0.0014)	0.8300(0.0000)	0.8227(0.0001)	**0.8406(0.0000)**
Average	0.3498(0)	0.7173(0.0032)	0.7833(0.0004)	0.7877(0.0048)	0.8337(0.0001)	0.8041(0.0001)	**0.8712(0.0002)**

The data in the **boldface** are the significantly best (or comparable best) results among these comparing methods, and the significance is checked by pairwise *t*-test at the 95% significance level. The average means the average RI of each method on eight gene expression datasets.

**Table 3:** ARI (average and standard deviation) of HC, *k*-means, SOM, LAC, WSPA, RDCFCE and EM-PCE on eight gene expression datasets.

Dataset	HC	*k*-means	SOM	LAC	WSPA	RDCFCE	EM-PCE
Breast	0.0565(0)	0.2492(0.0118)	0.2616(0.0008)	0.132(0.0028)	0.2110(0.0029)	0.2287(0.0023)	**0.3909(0.0040)**
DLBCLA	0.0034(0)	0.1391(0.0002)	0.2216(0.0000)	0.7831(0.0234)	0.6567(0.0023)	0.3440(0.0000)	**0.8839(0.0002)**
Leukemia	0.0015(0)	0.7909(0.0158)	0.6298(0.0271)	0.7858(0.0183)	0.5703(0.0034)	0.4779(0.0023)	**0.9158(0.0041)**
NovartisBPLC	0.3184(0)	0.4860(0.0085)	0.6512(0.0009)	0.7524(0.0268)	0.8954(0.0005)	**0.9463(0.0000)**	**0.9463(0.0000)**
Pomeroy2002v2	0.1012(0)	0.4720(0.0019)	0.4744(0.0040)	0.3010(0.0232)	**0.6296(0.0093)**	0.4657(0.0005)	0.4253(0.0023)
Ramaswamy2001	-0.0029(0)	0.1101(0.0009)	0.1973(0.0003)	0.1535(0.0057)	**0.4616(0.0060)**	0.2145(0.0017)	0.3936(0.0050)
Risinger2003	-0.0992(0)	0.1173(0.0151)	0.2501(0.0005)	0.0680(0.0057)	0.2752(0.0020)	**0.3904(0.0007)**	0.3171(0.0041)
Su2001	0.0104(0)	0.1332(0.0005)	0.1360(0.0003)	0.1386(0.0027)	0.1962(0.0006)	0.1681(0.0005)	**0.2062(0.0001)**
Average	0.0.0600(0)	0.3122(0.0007)	0.3528(0.0042)	0.3916(0.0136)	0.4870(0.0034)	0.4045(0.0010)	**0.5599(0.0025)**

The data in the **boldface** are the significantly best (or comparable best) results among these comparing methods, and the significance is checked by pairwise *t*-test at the 95% significance level. The average means the average ARI of each method on eight gene expression datasets.

**Table 4:** NMI (average and standard deviation) of HC, *k*-means, SOM, LAC, WSPA, RDCFCE and EM-PCE on eight gene expression datasets.

Dataset	HC	*k*-means	SOM	LAC	WSPA	RDCFCE	EM-PCE
Breast	0.1636(0)	0.4082(0.0119)	0.4086(0.0013)	0.3446(0.0036)	0.2877(0.0008)	0.4001(0.0025)	**0.5408(0.0054)**
DLBCLA	0.0295(0)	0.2008(0.0003)	0.2513(0.0006)	0.7958(0.0144)	0.5794(0.0005)	0.3708(0.0000)	**0.8525(0.0005)**
Leukemia	0.0368(0)	0.8221(0.0002)	0.7160(0.0062)	0.8656(0.0020)	0.6697(0.0016)	0.6706(0.0000)	**0.9140(0.0006)**
NovartisBPLC	0.5631(0)	0.6541(0.0160)	0.6648(0.0005)	0.8066(0.0072)	0.8885(0.0005)	**0.9495(0.0000)**	0.9400(0.0000)
Pomeroy2002v2	0.3795(0)	0.6070(0.0008)	0.6055(0.0033)	0.3847(0.0177)	**0.7423(0.0012)**	0.5842(0.0000)	0.5916(0.0008)
Ramaswamy2001	0.1123(0)	0.4998(0.0009)	0.5309(0.0002)	0.4569(0.0012)	**0.6308(0.0000)**	0.4949(0.0000)	0.6036(0.0005)
Risinger2003	0.0992(0)	0.2801(0.0034)	0.3918(0.0002)	0.2956(0.0076)	0.3845(0.0010)	**0.4712(0.0002)**	0.4163(0.0007)
Su2001	0.1357(0)	0.3119(0.0013)	0.3192(0.0005)	0.3604(0.0001)	**0.4232(0.0001)**	0.3865(0.0006)	0.4157(0.0001)
Average	0.1900(0)	0.4730(0.0044)	0.4860(0.0016)	0.5388(0.0045)	0.5758(0.0007)	0.5410(0.0004)	**0.6593(0.0010)**

The data in the **boldface** are the significantly best (or comparable best) results among these comparing methods, and the significance is checked by pairwise *t*-test at the 95% significance level. The average means the average NMI of each method on eight gene expression datasets.

HC constantly merges the closest samples into a new cluster, but the similarity between samples becomes isometric when a larger number of genes are involved and the similarity can be further distorted by noisy genes. Therefore, it frequently loses to other comparing methods. For the same reason, *k*-means also does not group samples into clusters as well as that of other comparing methods. We can see that LAC has similar performance with SOM. WSPA and RDCFCE have higher averages and smaller variances than SOM and LAC on most datasets. It is obvious that ensemble clusterings achieve higher accuracy and are more stable than single clustering algorithms. EM-PCE shows better performance on six datasets than RDCFCE under both RI and NMI, and shows better performance on five datasets than RDCFCE under ARI. The improvement is 8.34% (for RI on average), 38.41% (for ARI on average) and 21.87% (for NMI on average). The possible reasons are as follows: (i) RDCFCE uses SOM to map high-dimensional gene expression data to a low dimensional grid, without explicitly considering irrelevant genes. In contrast, EM-PCE obtains base clustering solutions by repeatedly running LAC, which gives weight to genes to reduce interference of irrelevant genes, and it can find a set of samples that have similar expression profiles only over a subset of genes. (ii) EM-PCE takes advantage of information from both sample-to-cluster assignments and gene-to-cluster assignments of multiple projective clustering solutions, but RDCFCE only regards to sample-to-cluster assignment. (iii) EM-PCE employs EM [49] to achieve the optimal sample-to-cluster assignment and gene-to-cluster assignment. RDCFCE gets the similarity of two samples by averaging sample-to-cluster assignments, and it does not distinguish the quality of base clustering solutions.

We also compare the performance of EM-PCE with WSPA. Both EM-PCE and WSPA use LAC as the base clustering. WSPA calculates the similarity of two samples based on a weighted distance of a sample to its corresponding cluster. From Tables 2–4, we can see that EM-PCE outperforms WSPA on seven out of eight datasets under RI, six out of eight datasets under ARI and five out of eight datasets under NMI. The improvement on average is 4.50% (RI), 14.97% (ARI) and 14.50% (NMI). The cause is that EM-PCE additionally takes gene-to-cluster into account in fusing multiple projective clusterings. In contrast, WSPA only takes into account sample-to-cluster assignment. In summary, these results demonstrate that projective clustering and ensemble clustering should be combined together to accurately cluster gene expression data, and EM-PCE can integrate the advantage of these two kinds of clustering techniques.

**Fig 3:**
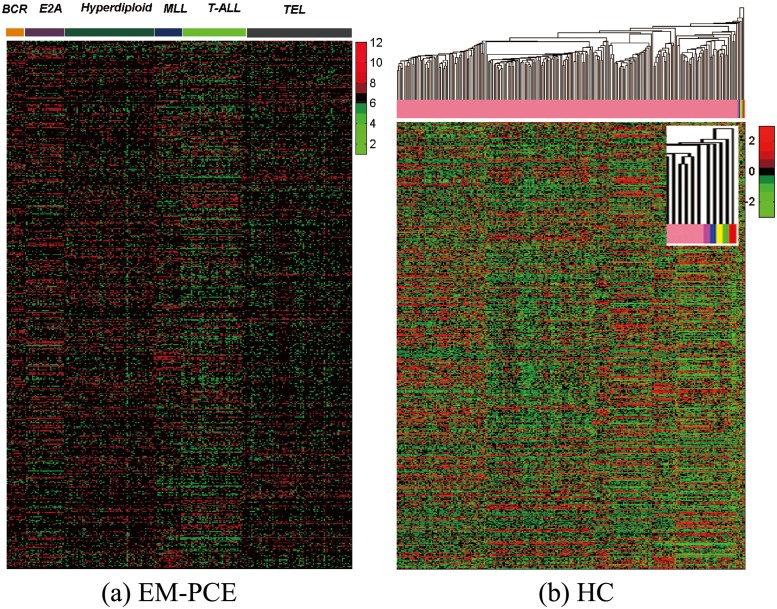
Heatmap of the clusters discovered by EM-PCE and HC on Leukemia cancer gene expression dataset. Leukemia cancer gene expression dataset contains 248 samples and are grouped into six subtypes (BCR, E2A, Hyperdiploid, MLL, T-ALL, TEL). Genes listed are the first 586 genes with the largest variances. Different clusters (subtypes) are marked by different color bars.

In addition, we also use heatmap to visually investigate the clusters discovered by EM-PCE and HC. Fig 3 shows the clustering result of EM-PCE and HC on Leukemia dataset, respectively. From the left sub-figure of Fig 3, we can see that the clusters (or subtypes) of Leukemia discovered by EM-PCE exhibit different gene expression profiles across genes, these clusters (named in the color bar) are in accordance with the ground truth subtypes. Although HC can also identify six clusters, but with one big cluster and five small clusters, which are not in accordance with the ground truth subtypes of Leukemia. In practice, HC can be cut off at any branch of tree to produce any number of clusters, we just choose to cut the tree to produce six clusters. Since these five small clusters are too small, we magnify the color bars corresponding to these five clusters to more clearly display them in Fig 3. We calculate the purity (PU) of the discovered clusters by EM-PCE and HC, $PU\left(C,C^{\prime }\right)=\frac{1}{n}\sum_{i=1}^{k}\underset{j\in \{1,...,k^{\prime }\}}{max}\left|c_{i}\cap c_{j}^{{}^{\prime }}\right|$, a larger value of PU means a better clustering result, the PU of EM-PCE is 0.960 and that of HC is 0.340. The visual results in Fig 3 and the PU measure again verify that EM-PCE is effective for clustering cancer subtypes, and also show HC is not a good option for clustering high-dimensional gene expression data. This observation corroborates the advantage of integrating gene-to-cluster assignment with sample-to-cluster assignment for gene expression data analysis. To make a clear heatmap, we select 586 genes with the largest variances of gene expression profiles from 985 genes across 248 samples.

### Sensitivity analysis

**Fig 4:**
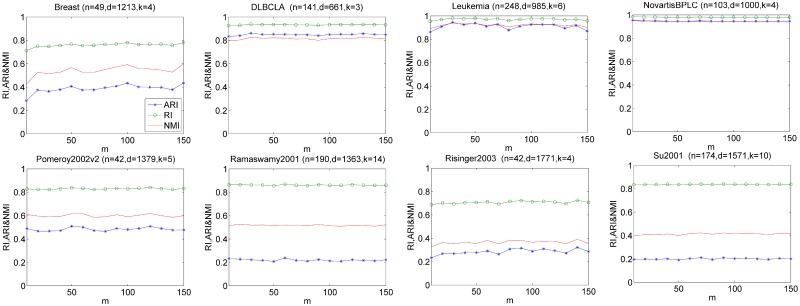
Sensitivity of m. For each *m*, we perform ten independently runs and report the average of RI and NMI. EM-PCE is robust to the input value of *m*.

In this section, we investigate the sensitivity of EM-PCE with respect to *m* (the number of base projective clusterings) and *α* (controlling the softness of sample-to-cluster assignments). We perform ten independent runs for each input value of *m* (or *α*) on eight datasets and report the average of RI, ARI and NMI. To study the performance of EM-PCE under different input values of *m*, we increase *m* from 10 to 150 and fix *α* = 2, EM-PCE generates base clustering solutions by repeatedly running LAC with *h* = 2. Fig 4 reports the results with respect to RI, ARI and NMI on eight datasets. From Fig 4, we can observe that RI, ARI and NMI are relatively stable on most datasets. Although, EM-PCE has fluctuation on Breast, the fluctuation is relatively small. The experimental results indicate EM-PCE is robust to input values of *m*. We suggest the *m* should set relatively large.

**Fig 5:**
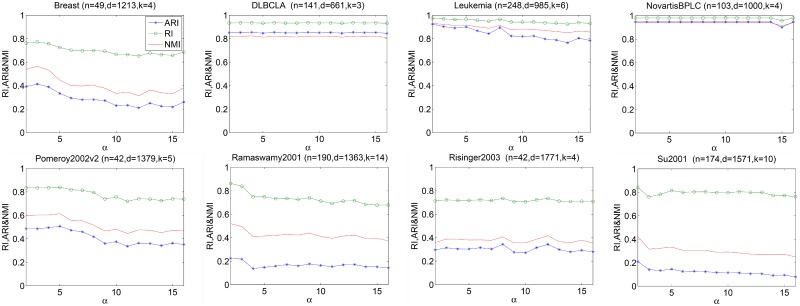
Sensitivity of *α*. For each *α*, we perform ten independently runs and report the average of RI, ARI and NMI.

Similarly, to investigate the sensitivity of EM-PCE to *α* (*α* > 1 is an integer parameter), we increase *α* from 2 to 16 and fix *m* = 100. EM-PCE generates base clustering solutions by repeatedly running LAC with *h* = 2. Fig 5 reports the results with respect to RI, ARI and NMI on eight datasets. From Fig 5, we can see that the accuracy of EM-PCE decreases when *α* is too large. So we suggest that *α* should not set too large, we set *α* = 2 in our experiments.

**Fig 6:**
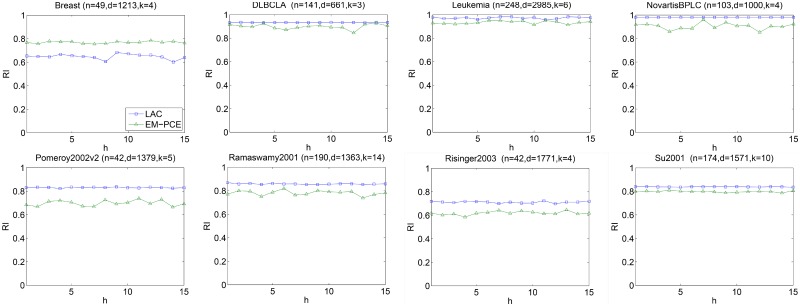
Sensitivity of h under RI. For each *h*, we perform 10 independently runs of LAC and EM-PCE under a particular value of *h*, and then report the average RI of LAC and EM-PCE, respectively.

**Fig 7:**
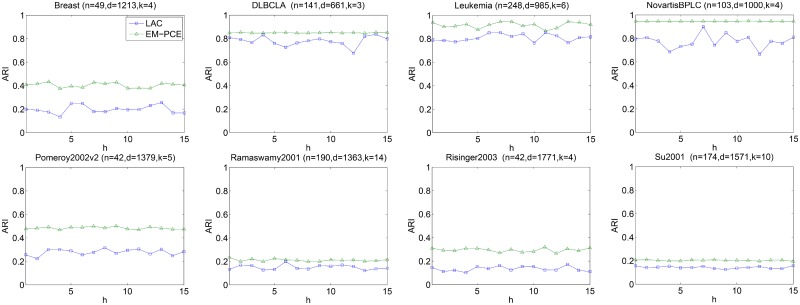
Sensitivity of h under ARI. For each *h*, we perform 10 independently runs of LAC (EM-PCE) under a particular value of *h* and report the average ARI of LAC and EM-PCE, respectively.

**Fig 8:**
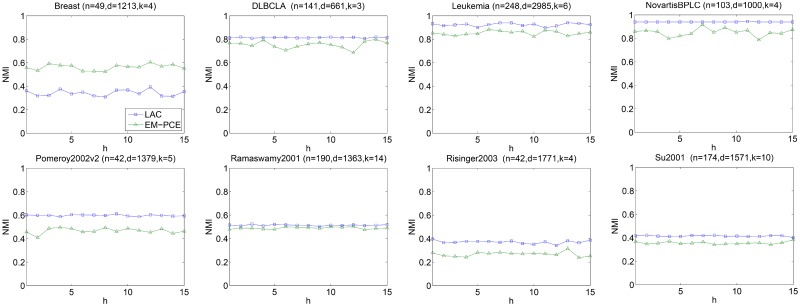
Sensitivity of h under NMI. For each *h*, we perform 10 independently runs of LAC (EM-PCE) under a particular value of *h* and report the average NMI of LAC and EM-PCE, respectively.

We also investigate the sensitivity of parameter *h* of LAC, since EM-PCE adopts LAC as base clustering. *h*(*h* > 0) controls the relative differences between gene weights. We vary *h* from 1 to 15, repeat LAC under each particular input value of *h* for 10 times and report the average results in Figs 6–8. As well as that, we repeat EM-PCE 10 times under a particular value of *h* and plot the average results in Figs 6–8. *α* is fixed as 2 and *m* is set as 100 in these experiments.

Figs 6–8 plot the results of LAC and EM-PCE with respect to RI, ARI and NMI under different input values of *h*. We can see that LAC is unstable on these eight datasets. LAC is sensitive to the input values of *h*. In contrast, EM-PCE not only has better results than LAC, but also is robust to *h*. The sensitivity analysis corroborates that single clustering algorithms often lack of stability and suffer from inappropriate setting of parameters. In contrast, ensemble clustering algorithms not only show more stable results, but also are more robust to input values than single clustering algorithms.

### Time complexity and runtime cost analysis

EM-PCE generates base clustering solutions by repetitively running LAC. LAC needs to iteratively optimize the weight assigned to genes for each cluster. Suppose the number of iterations for LAC to converge is *t*1, the time complexity of LAC is *O*(*t*1 × *k* × *n* × *d*), where *k* is the number of clusters, *n* is the number of involved samples and *d* is the number of genes. Therefore, the time complexity of generating *m* base LAC clustering solutions is *O*(*m* × *t*1 × *k* × *n* × *d*). EM-PCE consists of another two parts. The first part computes $\Lambda_{n^{\prime },d^{\prime }}=\frac{1}{m}\sum_{l=1}^{m}\sum_{k^{\prime }=1}^{k}X_{k^{\prime },n^{\prime }}^{l}Y_{k^{\prime },d^{\prime }}^{l}$, and the time complexity is *O*(*m* × *k*). For *d* genes and *n* samples, the time complexity of the first part comes to *O*(*m* × *k* × *n* × *d*). Another part of EM-PCE is to iteratively compute X* and Y* until convergency. Suppose the number of iterations for EM-PCE to converge is *t*2, and the total time complexity of this part is *O*(*k* × *n* × *d* × *t*2). In summary, the overall time complexity of EM-PCE is *O*(*k* × *n* × *d* × (*t*2 + *t*1 × *m*)).

**Fig 9:**
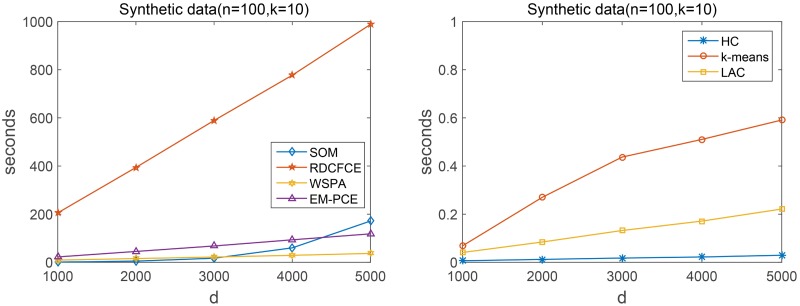
Runtime costs (seconds) on synthetic datasets. *d* is the number of genes. The dataset contains 100 samples from 10 clusters. Since HC, *k*-means and LAC run much faster than other comparing methods, we report the runtime costs of these comparing methods in two separate figures for better visualization.

We record the runtime costs of EM-PCE and other comparing methods, and reveal the results in Table 5. All the comparing methods are implemented with Matlab2012b and the experimental platform is: Windows 7, 8GB RAM, Intel(R) Core(TM) i5-4590. In order to study the runtime cost more intuitively, we also apply these comparing methods on synthetic datasets. We fix the number of samples as 100 and increase the number of genes from 1000, 2000, …, 5000. Fig 9 gives the runtime costs of these methods on synthetic datasets. From Table 5 and Fig 9, it is easy to observe that single clustering algorithm (HC, *k*-means, LAC, SOM) runs much faster than other comparing methods. The runtime of RDCFCE increases rapidly when the number of genes increasing and it takes more time than all the other comparing methods. That is because RDCFCE repeats SOM multiple times to find representative genes and then applies a fuzzy extension model on representative genes found by each SOM to generate multiple base clusterings. EM-PCE takes more time than WSPA. The reason is that EM-PCE not only has to run LAC multiple times to generate base clusterings, but also to optimize the sample-to-cluster assignment and gene-to-cluster assignment. WSPA only optimizes the sample-to-cluster assignment, so it takes fewer time than EM-PCE. The runtime of WSPA and EM-PCE increases relatively slow, and is even smaller than single clustering algorithm SOM when the number of genes becoming large. Given the superior results of EM-PCE with respect to these competitive algorithms, we can conclude EM-PCE is an effective alternative technique for clustering cancer gene expression data.

**Table 5:** Runtime cost (seconds) on real cancer gene expression dataset.

Dataset	HC	*k*-means	SOM	LAC	WSPA	RDCFCE	EM-PCE
BreastB	0.03	0.23	12.57	0.50	101.80	2510.98	302.70
DLBCLA	0.09	0.24	4.96	0.58	117.63	1946.50	236.34
Leukemia	0.33	0.90	13.10	3.28	412.93	4722.94	1144.20
NovartisBPLC	0.07	0.19	8.10	0.61	87.44	2172.79	354.17
Pomeroy2002v2	0.03	0.23	21.18	0.36	40.01	1381.63	217.04
Ramaswamy2001	0.28	2.21	30.93	5.51	631.94	5248.20	2747.36
Risinger2003	0.04	0.23	70.00	0.43	43.56	1727.51	228.74
Su2001	0.27	1.73	36.12	5.16	466.25	5491.07	2098.11
Overall	1.14	5.98	196.97	16.44	1910.56	10331.31	7328.66

The runtime costs of HC, *k*-means, SOM, LAC, WSPA, RDCFCE and EM-PCE on eight real gene expression datasets. RDCFCE costs more time than other methods.

## Conclusion

In this paper, we investigate EM-PCE for clustering cancer gene expression data. EM-PCE leverages the advantage of projective clustering to handle high dimensional gene expression data and utilizes the merits of ensemble clustering to produce stable clustering solution. Experimental results show that EM-PCE outperforms other related approaches on clustering gene expression data and is robust to the noise. The parameter sensitivity study also shows EM-PCE is robust to input parameters. These comparative results demonstrate that EM-PCE is more promising to discover cancer subtypes. EM-PCE can be adopted to identify functionally correlated expression patterns and explore bi-clusters from high-dimensional gene expression data. Given the nature of gene expression data, we will investigate more efficient and effective co-clustering ensemble algorithms for gene expression data analysis.
